# Characterizing Depth of Defects with Low Size/Depth Aspect Ratio and Low Thermal Reflection by Using Pulsed IR Thermography

**DOI:** 10.3390/ma14081886

**Published:** 2021-04-10

**Authors:** Alexey I. Moskovchenko, Michal Švantner, Vladimir P. Vavilov, Arsenii O. Chulkov

**Affiliations:** 1 New Technologies Research Centre, University of West Bohemia, Univerzitní St. 2732/8, 301 00 Plzeň, Czech Republic; alexeym@ntc.zcu.cz (A.I.M.); msvantne@ntc.zcu.cz (M.Š.); 2 Engineering School of Nondestructive Testing, Tomsk Polytechnic University, 30 Lenin Avenue, 634050 Tomsk, Russia; chulkovao@tpu.ru; 3 Faculty of Mechanics and Mathematics, Tomsk State University, 30 Lenin Avenue, 634050 Tomsk, Russia

**Keywords:** pulse thermography, defect aspect ratio, thermal reflection coefficient, thermal NDT, defect characterization, non-linear fitting, thermographic signal reconstruction

## Abstract

This study is focused on the quantitative estimation of defect depth by applying pulsed thermal nondestructive testing. The majority of known defect characterization techniques are based on 1D heat conduction solutions, thus being inappropriate for evaluating defects with low aspect ratios. A novel method for estimating defect depth is proposed by taking into account the phenomenon of 3D heat diffusion, finite lateral size of defects and the thermal reflection coefficient at the boundary between a host material and defects. The method is based on the combination of a known analytical model and a non-linear fitting (NLF) procedure. The algorithm was verified both numerically and experimentally on 3D-printed polylactic acid plastic samples. The accuracy of depth prediction using the proposed method was compared with the reference characterization technique based on thermographic signal reconstruction to demonstrate the efficiency of the proposed NLF method.

## 1. Introduction

Past decades demonstrate a growing popularity of infrared (IR) thermography as an established nondestructive testing (NDT) technique, particularly as used in the aerospace industry [1,2,3], power production [4,5], civil engineering [6,7,8] and manufacturing of composite and hybrid materials [9,10,11], thanks to its high inspection productivity, illustrative character of data presentation and sensitivity to defects of various origins. It is often stated that IR thermographic NDT is a perfect method for the detection and evaluation of shallow laterally-extended defects, such as disbonds, delaminations, corrosion material loss, etc. Known limitations of this method are related to the detection of deep- and small-size defects, and difficulties of defect characterization that is conditioned by heat diffusion in bulk materials.

Under the term “defect characterization”, one understands the quantitative evaluation of material thermal properties, defect depth, lateral size and thermal resistance. Determining defect depth and size is the most important characterization procedure for the assessment of quality and the lifetime of responsible parts and components, including the evaluation of necessary repair size.

There are plentiful publications devoted to quantitative depth evaluation [12,13,14,15,16,17]. Many studies establish the proportionality between squared defect depths and the corresponding specific characteristic time (SCT) of heat conduction. The time of maximal temperature differential signals (peak contrast), time of maximal logarithmic derivatives or some specific parameters obtained by special transformations of temperature can be considered as SCTs [18]. Marinetti et al. proposed analyzing temperature evolutions in the frequency domain and using the zero-crossing frequency for determining defect depth [19]. All above-mentioned methods are based on a 1D heat conduction model, thus being suitable for evaluating laterally-extended defects characterized by a high size/depth aspect ratio, i.e., the so-called 1D defects. In other words, the suggested depth inversion formulas do not account for the size of defects and the influence of 3D heat diffusion. Long SCT values correspond to intensive “smashing” of temperature signals because of heat diffusion, thus leading to greater inversion inaccuracies. The characterization accuracy improves with shorter observation times when lateral heat diffusion is “weak”. For instance, the so-called “early observation time” [20] and a peak time of the second logarithmic derivative [21] allow for the prediction of the depth of relatively small defects due to the 1D character of heat conduction. However, such an approach is limited by amplitudes of temperature signals and the influence of heat pulse duration.

Almond and Pickering [22] modified a 1D analytical model in order to take into account a finite size of defects, and they used it for the prediction of peak contrast amplitudes and times for defects with different aspect ratios. The model assumed that the effective thermal reflection coefficient at the solid-air interface *R* = 1 that is not true in many practical cases. It was demonstrated in [23] that the magnitude of the thermal reflection coefficient in a carbon fiber reinforced composite (CFRP) sample with flat bottom holes (FBH) varies between 0.18 and 0.84, depending on FBH depth. This suggests that the real defects, such as inclusions, delaminations, cracks etc., should be characterized by varying *R* values. A weak dependence of SCT on *R* was demonstrated in [17,23] without taking into account defect finite dimensions. In fact, the model suggested in [22] shows that *R* values affect both the amplitude and time of peak contrasts, as well as the shape of temperature evolution curves and, correspondingly, the values of other proposed SCTs.

Since the SCT methods are not fully appropriate for characterizing deeper defects with low size/depth aspect ratio, a non-linear (least-square) fitting (NLF) method was developed [23,24]. The method involves the fitting of experimental temperature evolutions with some theoretical models by implementing known curve fitting tools. A new NLF technique introduced in [23] took into account a non-unity thermal reflection coefficient, but not 3D heat diffusion, thus limiting characterization accuracy.

In this study, a novel method for estimating depth of defects with a low defect size/depth ratio, i.e., taking into account the 3D heat diffusion phenomenon, was proposed. The method is based on the combination of a known analytical model and a NLF algorithm. It was verified by performing both numerical modeling and experimentation on 3D printed samples with embedded defects. Unlike the above-mentioned approaches, the proposed methodology considers the finite size of defects and introduces an effective thermal reflection coefficient, which is less than unity, thus taking into account thermal properties of both a tested material and defects. In addition, defects of different geometrical shape, such as disks and spheres (air bubbles), were analyzed in comparison to commonly used flat bottom holes (FBHs).

## 2. Theory

A temperature response of an adiabatic semi-infinite body subjected to flash heating is described with the well-known expression [25]

(1) $$T\left(0,t\right)=\frac{Q_{0}}{\sqrt{\pi \rho Ckt}}=\frac{Q_{0}}{e\sqrt{\pi t}}$$

where *T*(0, *t*) is the surface temperature on the surface (*x* = 0) at the time *t* after flash heating with the energy intensity $Q_{0}$, and *ρ*, *C*, *k* and *e* are the material density, specific heat capacity, thermal conductivity and thermal effusivity (*e* =$\;\sqrt{\rho Ck}$), respectively. A sample area containing a thermally insulated defect at the depth *d* may be replaced with a slab with thickness *d*. The temperature response of such a slab is as follows [26]:

(2) $$T\left(0,t\right)=\frac{Q_{0}}{e\sqrt{\pi t}}\left[1+2\overset{\infty }{\underset{n=1}{\sum }}R^{n}e^{-\frac{{\left(nd\right)}^{2}}{\alpha t}}\right]$$

where α is the thermal diffusivity, *R* is the thermal reflection coefficient (contrast of thermal effusivities) and *n* is the summation index. The differential temperature signal contrast between the defect and non-defect areas, often called thermal contrast, may be obtained by subtracting Equation (2) from Equation (1):

(3) $$T\left(0,t\right)=\frac{2Q_{0}}{e\sqrt{\pi t}}\left[\overset{\infty }{\underset{n=1}{\sum }}R^{n}e^{-\frac{{\left(nd\right)}^{2}}{\alpha t}}\right]$$

**Figure 1:**
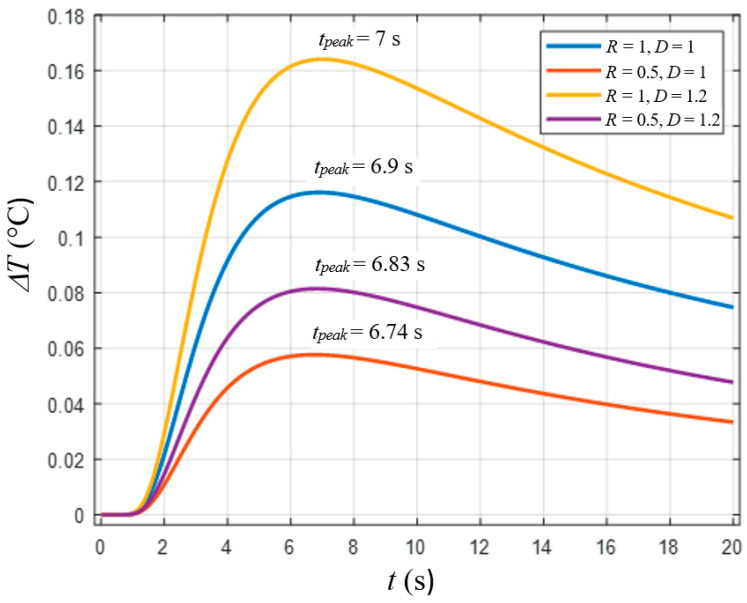
Differential temperature signal (Δ*T*) curves vs. time for different values of thermal reflection coefficient *R* and defect diameter *D* (obtained by Equation (4), *Q*_0_ = 10,000 J/m^2^, *e* = 538 W·s^1/2^·m^−2^·K^−1^, *α* = 0.58·10^−7^ m^2^/s, *d* = 1 mm, *m* = 1, *n* = 10).

In order to account for defect size and thermal diffusivity anisotropy, Almond and Pickering introduced a decay term into Equation (3) considering a delamination edge as a heat sink [22]. The expression for the surface temperature signal ΔT over the center of a circular defect was suggested in the form

(4) $$\Delta T\left(0,t\right)=\frac{2Q_{0}}{e\sqrt{\pi t}}\left[\overset{\infty }{\underset{n=1}{\sum }}R^{n}e^{-\frac{{\left(nd\right)}^{2}}{\alpha t}}\right]\left(1-e^{-\frac{D^{2}}{16m\alpha t}}\right)$$

where *D* is the diameter of a defect and *m* is a ratio of in-plane and through-thickness diffusivities of the material. Equation (3) was used for determining detection limits but assuming *R* = 1 and *n* = 1, which might not be true in practice [23]. According to [17,23], the analysis of Equation (3) shows that *R* values mainly affect maximal temperature signals (peak contrasts) but not optimal observation times (peak contrast times *t_peak_*). However, it follows from Equation (4) that a change in the coefficient *R* also shifts a position of the peak contrast time if the finite size of defects is taken into account. This process is demonstrated in Figure 1, where ΔT evolutions of for different reflection coefficients and defect diameters are presented. Because both Δ*T* and *t_peak_* values are influenced by 3D heat diffusion, the known methods of defect characterization by SCTs may be inaccurate. Therefore, it is suggested to apply a NLF technique to fit experimental data with the analytical solution by Equation (4).

It was shown in [22] that both experimental and simulated temperature evolutions can effectively be fitted with Equation (4) at the rising slope of Δ*T*(*t*) curves, while major discrepancies between fitted and original data occurred at longer times. Therefore, the time period preceding the peak contrast time has been chosen for fitting.

It is worth reiterating that the analytical models in thermal NDT are typically 1D, i.e., they mainly refer to planar defects oriented in a parallel direction with respect to the sample surface. In fact, the expressions above are approximate and allow for the correcting 1D analytical solutions by finite size of defect.

When applying a NLF technique, another challenge is the number of variables in Equation (4). Fitting many unknown parameters may result in a long computation time and worsened characterization accuracy. Fortunately, some variables can be evaluated experimentally. For example, thermal diffusivity can be determined with a fairly high accuracy by using the Parker method [27,28]. Defect lateral size is the only parameter, which can be evaluated visually by surface defect indications or use of other techniques [29], and this issue is beyond the scope of this study. However, evaluation of thermal conductivity and heat capacity requires measuring input energy that is difficult without using special equipment. In Equation (4), these two parameters appear together, and they can be replaced by the so-called apparent effusivity [30,31]:

(5) $$e_{app}\left(t\right)=\frac{1}{T\left(t\right)\sqrt{\pi t}}$$

A theoretical value of *e_app_* can be obtained by combining Equations (1) and (5):

(6) $$e_{app}=\frac{e}{Q_{0}}=\frac{\sqrt{k\rho C}}{Q_{0}}$$

In this way, only two unknown parameters become candidates for the fitting: the defect depth *d* and the effective thermal reflection coefficient *R*. Then, the proposed model is described by the following formula:

(7) $$\bar{T}\left(R,d,D,e_{app},\;\alpha ,t\right)=\frac{2}{e_{app}\sqrt{\pi t}}\left[\overset{M}{\underset{n=1}{\sum }}R^{n}e^{-\frac{{\left(nd\right)}^{2}}{\alpha t}}\right]\left(1-e^{-\frac{D^{2}}{16m\alpha t}}\right)$$

where *M* is the number of iterations. In this study, it was assumed that *M =* 10, since the higher summing terms have appeared negligible. As mentioned above, the fitting was performed in the 0 < *t* < *t_peak_* time range, where *t_peak_* is the peak contrast time. The NLF algorithm used the Matlab-based lsqnonlin least-square solver to solve a five-parameter optimization problem. Note that three parameters, *D*, *e_app_* and *α*, were estimated independently on the basis of the experimental data and introduced into the algorithm within the corresponding ±10% intervals. The fitting procedure has been formulated as

(8) $$\underset{R,d,D,e_{app},\alpha }{\min}\|\Delta \bar{T}\left(t\right)-\Delta T\left(t\right)\|$$

where $\Delta T\left(t\right)=T_{d}-T_{nd}$ is the experimental temperature contrast between defect centers and adjacent non-defect areas. Values of *R* varied from −1 to 1, with the initial value being 1, while the defect depth d range was 0.1–10 mm (initial value was 1 mm).

## 3. Numerical Modeling

Numerical modeling was used to demonstrate efficiency of the proposed analytical model and NLF algorithm in characterizing defect depth in cases where 3D heat diffusion becomes considerable (low size/depth ratio) and the algorithms based on 1D models reveal low characterization accuracy. Additionally, the applicability of the model for fitting defects with different shapes, such as FBH, disk and sphere, by changing the thermal reflection coefficient *R*, was checked. The results of the numerical modeling were also compared with depth characterization results by applying the known technique of thermographic signal reconstruction (TSR), or a second time derivative method, which is assumed to be less affected by 3D diffusion phenomena than SCT methods [13].

**Figure 2:**
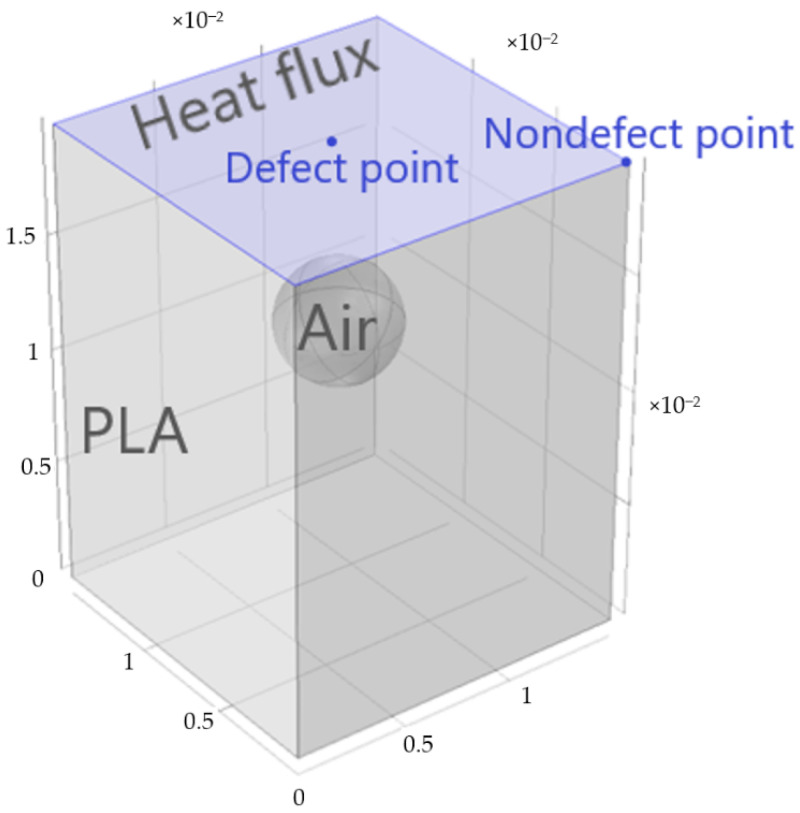
Numerical model of active thermal NDT (Comsol Multiphysics).

The numerical modeling was performed by using the Comsol Multiphysics software (version 5.0, Comsol, Stockholm, Sweden). The model simulated 3D heat conduction in a solid block (5 mm × 5 mm × 20 mm, 10 mm × 10 mm × 20 mm and 15 mm × 15 mm × 20 mm) with an air defect inside. Different shapes of defects were analyzed, such as FBH, disk and sphere. Three detect diameters *D* (1, 3 and 5 mm) and three depths corresponding to the size/depth ratios of 5, 2 and 1 were considered for all types of the defects. The depth was determined as the distance from the front sample surface to the upper border point of the defects. An example of the model is shown in Figure 2.

The pulsed heat flux (power density 100 kW/m^2^, duration 0.1 s) was delivered to the sample top (front) surface, and all other slab surface were adiabatic. The block material was polylactic acid plastics (PAL), and the thermal property values were borrowed from the published data [32] (PAL) and Comsol Multiphysics library (air), see Table 1.

**Figure 3:**
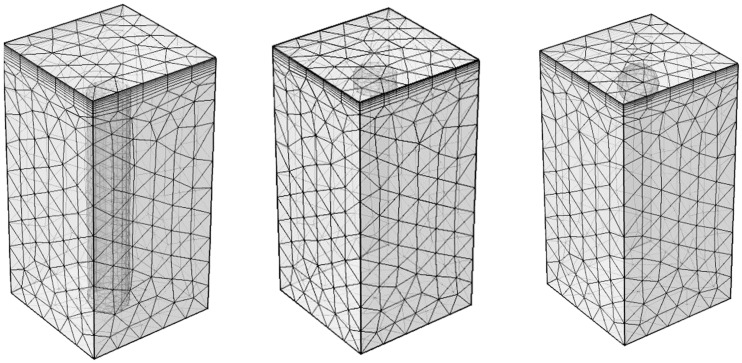
Examples of finite element mesh in Comsol Multyphisics (from left to right: FBH, disk, sphere).

The finite element mesh contained from 10,372 to 38,597 tetrahedron elements (more elements for smaller defects). An average size of each element was 2 mm; however, a boundary layer with elements of thickness about 0.1–0.3 mm was placed on the top surface. Examples of used meshes for different defect types are shown in Figure 3. A regular time step of 0.05 s was used in computations. Modeling results were presented as temperature evolutions at surface defect and non-defect points with the time interval of 0.05 s (Figure 2). The model included a “Heat transfer in solids” physical interface and a time-dependent solver. The temperature evolution was obtained by 1D cut points.

## 4. Experimental Setup and Samples

**Figure 4:**
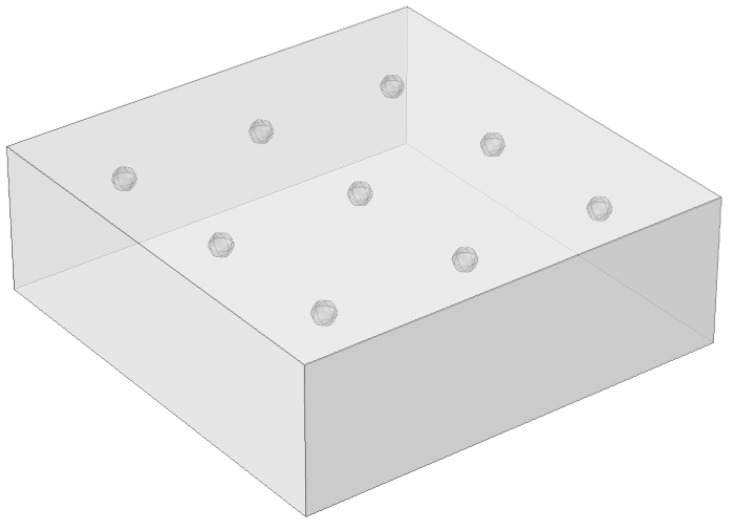
Scheme of sample with sphere-like defects (*D* = 3 mm).

3D printing technologies allow for the manufacturing samples of complicated geometries including defects, such as voids, inclusions, air bubbles, etc. Nine 60 mm × 60 mm × 20 mm PAL samples containing defects of three types (FBH, disk and sphere) with varying lateral size and depth were printed for experimentation. An example of the sample containing nine 3 mm-diameter sphere-like defects is shown in Figure 4, and the parameters of all sphere-like artificial defects are listed in Table 2; note that the defect depth is defined as the distance from the top surface of the sample to the top point of the defect. Additionally, a 60 mm × 60 mm × 2 mm defect-free sample was manufactured for evaluating PAL thermal diffusivity.

**Figure 5:**
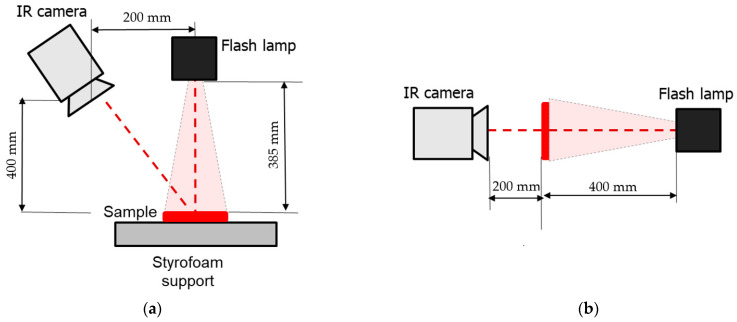
Thermal NDT procedures: (**a**) reflection mode, (**b**) transmission mode.

The experimental setup included a FLIR SC7650 IR (FLIR Systems, Wilsonville, OR, USA) camera and a Hensel EH Pro 6000 (Hensel, Fairfield, NJ, USA) flash tube (optical pulse energy 6 kJ, pulse duration 6 ms, distance to the sample 385 mm). The reflector of the flash tube was covered with a protective glass to cut spurious IR radiation. Image sequences consisting of 2500 IR thermograms (320 × 256 image format) were captured with a 50 Hz acquisition rate and 1.432 ms integration time. The schemes of one- (reflection mode) and two-sided (transmission mode) thermal NDT procedures are presented in Figure 5 (the setup of Figure 5b was used for determining diffusivity).

The time of the flash was synchronized with the recorded frames as described elsewhere [18]. Ten pre-frames were captured before starting the flash, and their average was subtracted from each image in the recorded sequences to deal with sample excess temperature only. In this way, the analyzed IR image sequences corresponded only to the sample cooling stage starting from the maximum temperature, which occurred at the end of the flash. Image acquisition was performed by using the LabIR software (version 1.1, University of West Bohemia, Praha, Czech Republic), while calculations of derivatives, apparent effusivity and defect lateral dimensions were fulfilled by means of the ThermoFit Pro software (version 5.6, Tomsk Polytechnic University, Tomsk, Russia). The used NLF algorithm was implemented on the MATLAB (version 8, Natic, MA, USA) platform.

## 5. Results and Discussion

### 5.1. Numerical Simulation

**Figure 6:**
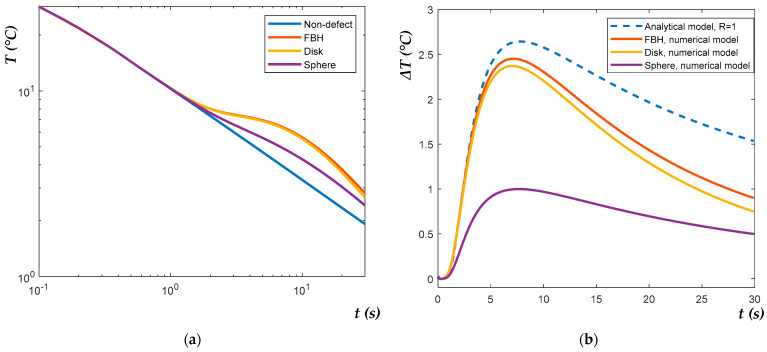
Temperature evolutions in logarithmic scale (**a**) and temperature contrasts Δ*T* (**b**) for FBH, disk and sphere (*D* = 3 mm and *d* = 0.6 mm) obtained by numerical modelling.

Figure 6a illustrates the results of numerical modeling for three types of defects (FBH, disk and sphere) with the same diameter (*D* = 3 mm) and depth (*d* = 0.6 mm). The temperature development in non-defect areas corresponds with the analytical solution for the semi-infinite body with the same thermal properties and represents the straight line with a slope of −0.5 on the logarithmic scale. For the defects, the temperature developments experience some deviations from the straight line, which are different for the different defect shapes (Figure 6a). This feature becomes more illustrative in the curves reflecting the contrasts, Δ*T(t)* = *T_d_*(*t*) − *T_nd_*(*t*); see Figure 6b. Figure 6b also shows the comparison between the numerical and analytical (by Equation (4)) values of Δ*T*(*t*) obtained for the identical test conditions; note the thermal reflection coefficient *R* = 1.

**Figure 7:**
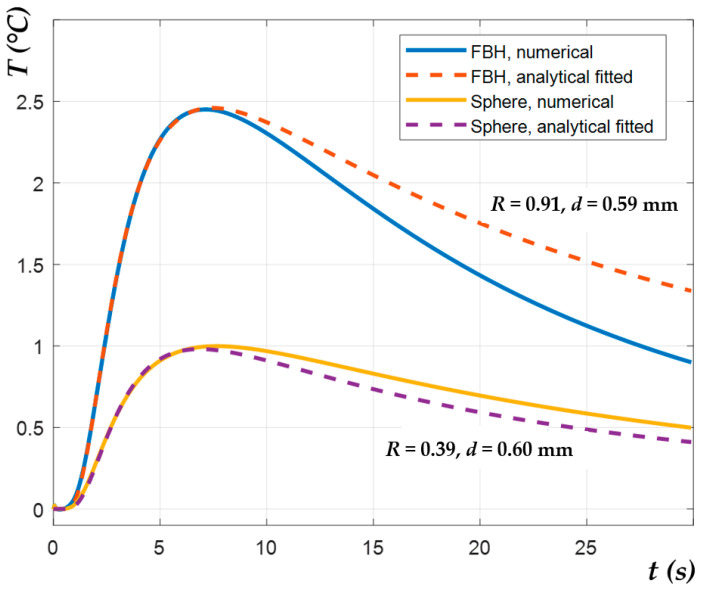
Evolutions of temperature contrasts Δ*T* for FBH and spheres with *D* = 3 mm and *d* = 0.6 mm obtained by numerical and analytical modelling with fitted *d* and *R* parameters.

Figure 6 demonstrates that, in a strict sense, defects of the same size but different shapes cannot be characterized by the same magnitude of the peak contrast Δ*T_max_* and peak contrast time *t_peak_*, independently, whether the test parameters are calculated numerically or analytically (by Equation (4)), and if the thermal reflection coefficient is assumed *R* = 1. It was supposed that modeling results can be improved by fitting *R* < 1 values and evaluating “effective” defect depths more accurately. Figure 7 illustrates applying the NLF algorithm (by Equation (8)) to the numerical data. Defect depth *d* and the thermal reflection coefficient *R* were set as unknown parameters to be fitted. The values *D*, *e_app_* and *α* were set the same as in the numerical model. As mentioned above, only the increasing slopes of the Δ*T(t)* signals, i.e., the time intervals *0 < t < t_peak_*, were analyzed, being individually determined for each Δ*T(t)* evolution curve.

**Figure 8:**
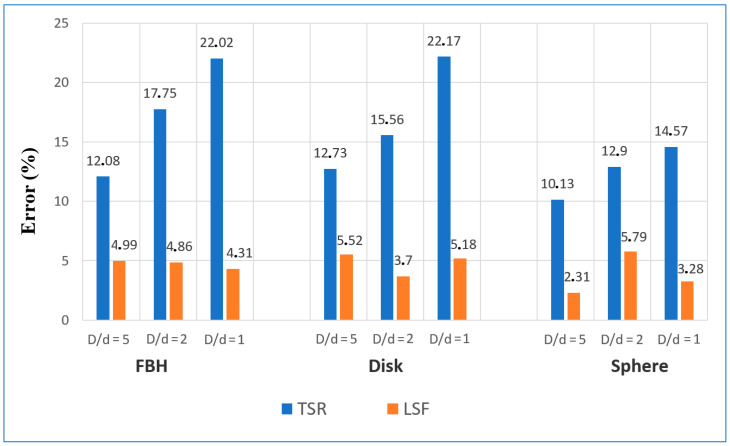
Mean relative errors of depth estimation by using TSR and NLF techniques (theory).

Within the considered time interval, the analytical model was found to fit very well to the numerical data. The depth values predicted by the NLF algorithm (analytical data) were compared with the results obtained by the second time derivative (Thermographic Signal Reconstruction–TSR) method applied to the numerical results [2]. The TSR technique was chosen for the comparison because it is weakly affected by 3D heat conduction, thus being very appropriate for evaluating low aspect ratio defects [33]. The comparison results are presented in Appendix A. However, the suggested NLF algorithm, which accounts for the defect size and thermal reflection at defect boundaries, revealed more accurate results of the defect characterization prediction than the TSR method. It is worth noting that the fitted values of *R* varied not only with defect shape but also with defect depth and diameter. In general, the thermal reflection coefficient was increasing with greater defect depths and diameters. Respectively, the *R* values in the case of FBH and disk-like defects (*R* = 0.7–1) were higher than those for spheres (*R* = 0.3–0.6), even if some deviations from this result were also observed. In fact, the proposed thermal NDT model is simplified and does not fully reflect the complexity of the 3D heat transfer phenomena in defect areas. Additionally, note that the *R* values retrieved by fitting do not represent true values of the thermal reflection between two materials, because they are affected by 3D heat conduction. In general, the results presented above illustrate the efficiency of the proposed approach for predicting defect depth in the case of low defect aspect ratios. Figure 8 illustrates the mean relative error of depth estimation *M* for the analyzed defects, with different aspect ratios obtained by the TSR and NLF methods. Here *M* is defined as

(9) $$M=\frac{1}{m}\overset{m}{\underset{n=1}{\sum }}\left|\frac{d_{t}-d_{p}}{d_{t}}\right|\times 100\%$$

where *d_t_* and *d_p_* are the true and predicted defect depths, and *m* = 3 is the number of measurements corresponding to the three diameters of the defects with the same aspect ratio.

The maximum relative errors of depth prediction by NLF method were about 12% and 11%, respectively, for the disk-like and FBH defects (*D* = 1 mm, *d* = 0.2 mm). In fact, such errors were expected because of the influence of a finite heat pulse that is particularly essential for shallow defects. However, in most cases, the errors did not exceed ±5–6% without a noticeable increase for defects with a higher aspect ratio (Figure 8). This implies that the suggested algorithm accounts for lateral size of defects and can be implemented for characterizing smaller defects. In turn, the relative error by using the TSR method has been significantly higher compared to NLF, and it grows with a lower defect aspect ratio. It is worth noting that the TSR technique has always supplied negative errors (Appendix A), i.e., the predicted defect depths have always been lower than the true depth values.

The results of the numerical modeling confirm validity of the earlier proposed analytical model, which considers 3D heat conduction phenomena occurring in defect areas. However, unlike many previous studies, the thermal reflection coefficient *R* cannot be assumed to be 1. The model is also useful in calculating temperature responses from sphere-like defects. In this case, the retrieved *R* values are lower than in the case of FBH defects. In general, the use of the NLF technique in combination with the analytical model seems to be more efficient at predicting defect depth compared to the TSR method.

### 5.2. Experimental Results

**Figure 9:**
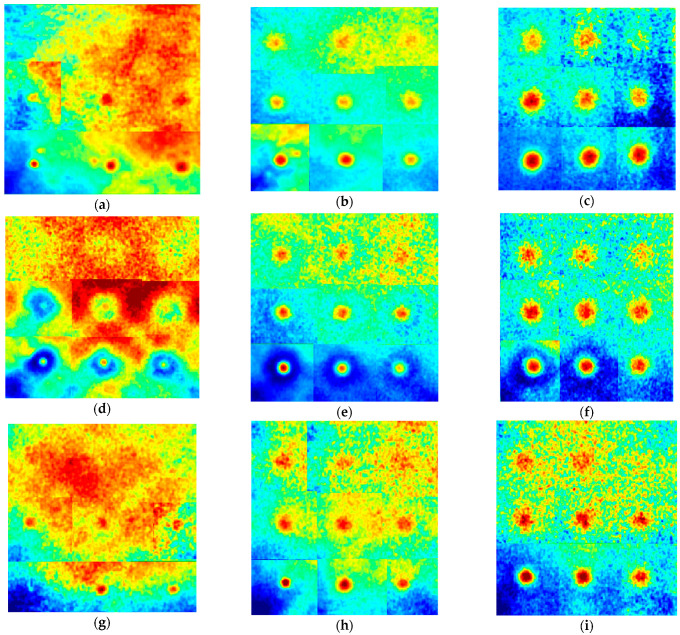
Synthetic IR thermograms of tested samples: (**a**) FBH, *D* = 1 mm, (**b**) FBH, *D* = 3 mm, (**c**) FBH, *D* = 5 mm, (**d**) disk, *D* = 1 mm, (**e**) disk, *D* = 3 mm, (**f**) disk, *D* = 5 mm, (**g**) sphere, *D* = 1 mm, (**h**) sphere, *D* = 3 mm, (**i**) sphere, *D* = 5 mm.

The experimental results revealed a fairly good visibility for all types of the defects. The maximum depths of reliably detected defects were 1.05, 2.4 and 3 mm for the defects with the diameter of 1, 3 and 5 mm, respectively. Figure 9 illustrates synthetic thermograms obtained by stitching the regions of interest for individual defects, which are characterized by different optimal observation times, as shown in Figure 7. In the thermal map of Figure 9, each defect indication has been cut from the corresponding raw “optimal” image to exhibit maximal Δ*T* signals in a single image.

Since the methodology used in this study is based on the analysis of Δ*T* evolutions, both defect and non-defect areas are to be identified. To suppress one-pixel signal spikes, 2 × 2 pixel areas were chosen in the centers of defect indications, and, to avoid the influence of uneven heating, 10 × 10 pixel non-defect areas were chosen close to each defect analyzed.

As noted above, the use of the NLF technique requires a priori knowledge of material diffusivity, apparent effusivity and defect lateral size.

Thermal diffusivity was determined by the Parker method on the 60 mm × 60 mm × 2 mm defect-free sample by the scheme described on p. 4 [27]. The sample material was considered isotropic by assuming the anisotropy coefficient *m* = 1. The determined diffusivity value averaged by the measurements at ten sample points and used in calculations was 1.04 × 10^−7^ m^2^·s^−1^.

**Figure 10:**
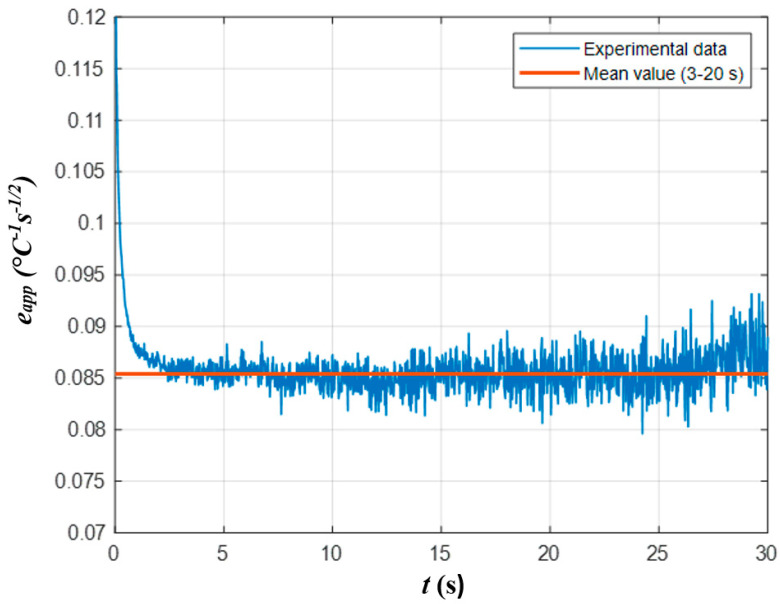
Apparent effusivity vs. time (PAL sample).

The apparent effusivity was computed by applying Equation (5) to experimental temperature developments, thus producing the sequences of apparent effusivity maps. Since apparent effusivity depends on absorbed energy *Q_0_*, only the effusivity magnitude in chosen reference areas was taken into account in calculations. For an adiabatic semi-infinite body heated by a Dirac pulse, the analytical function “apparent effusivity vs. time” represents a straight line parallel to the *x*-axis. However, the corresponding experimental functions are distorted by the influence of heat pulse duration and surface heat losses, as well as by spatial/temporal noises of various origins. Therefore, in this study, the apparent effusivity was taken as a mean value in a chosen reference area within the time interval of 3–20 s, where the retrieved apparent effusivity values are weakly affected by pulse duration and heat losses. Figure 10 shows the development of both theoretical and experimental apparent effusivity with time (time interval 3–20 s, FBH, *D* = 3 mm, *d* = 0.6 mm).

Reviewing techniques for determining defect lateral size is beyond the scope of this research; see [34,35]. In this study, the true defect dimensions were used as the mean values in the NLF algorithm within the ±10% interval.

**Figure 11:**
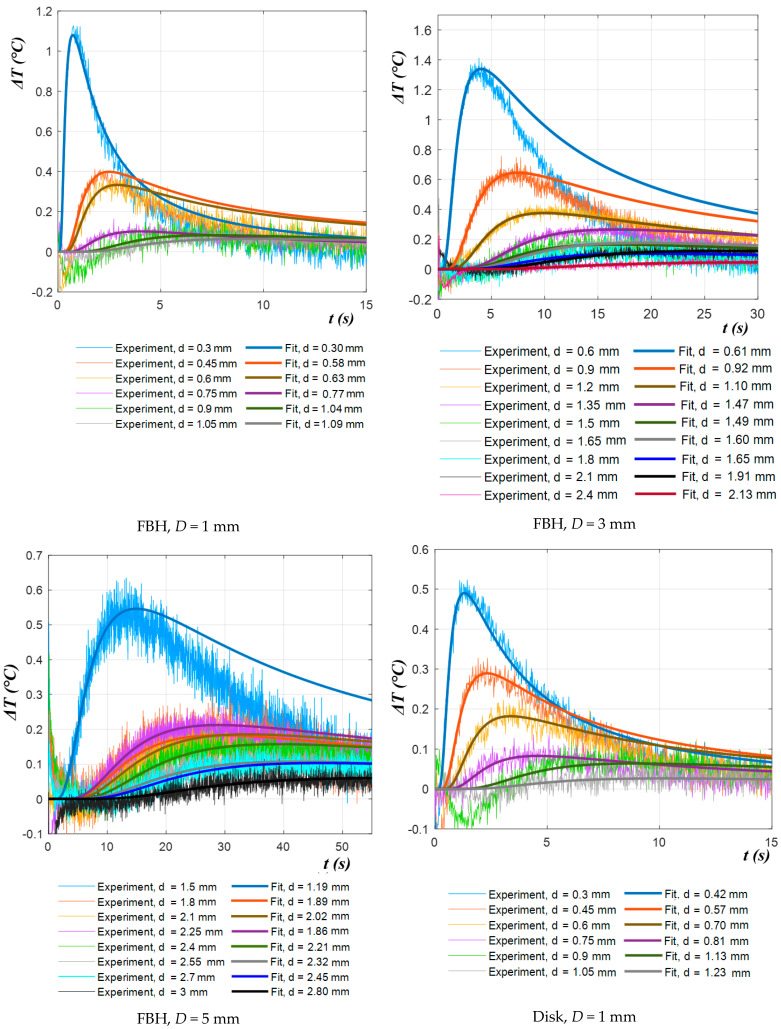

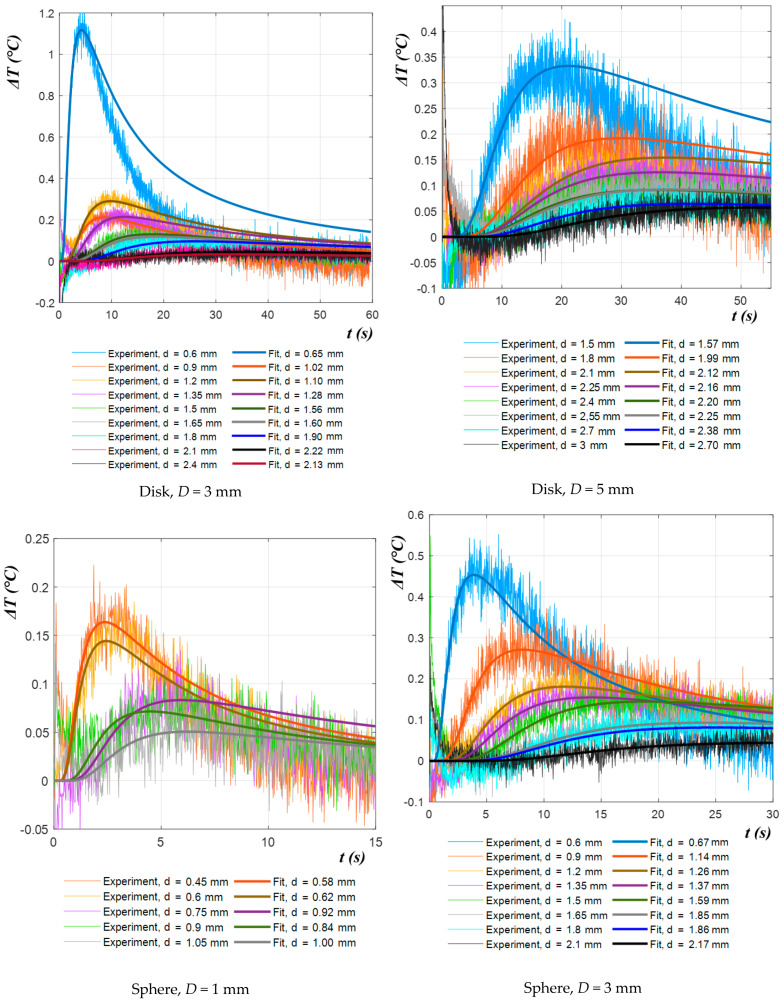

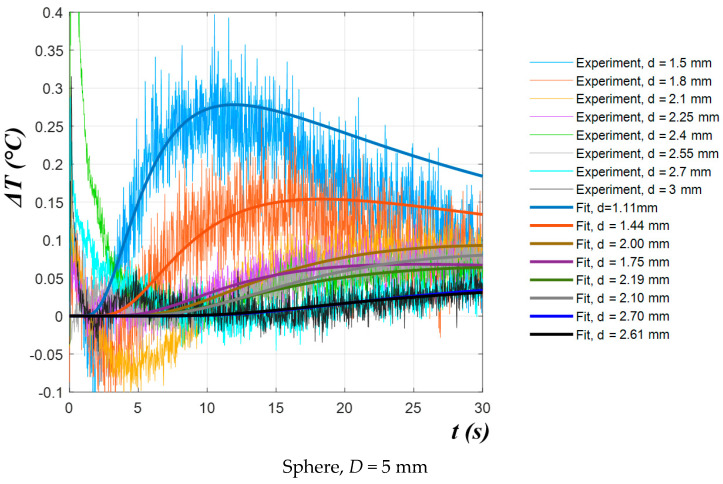
Experimental and fitted Δ*T* evolutions.

Figure 11 shows the temperature contrast (Δ*T*) curves obtained from the experimental data for each defect, along with the corresponding analytically-fitted curves obtained by applying the NLF algorithm.

**Figure 12:**
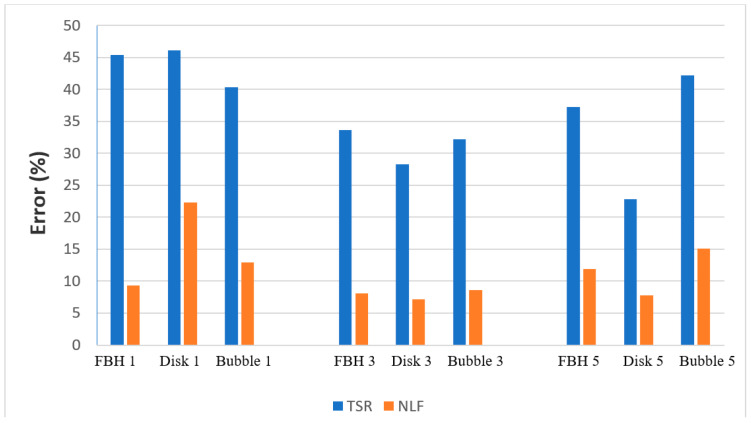
Mean relative errors of depth estimation by using TSR and NLF techniques (experiment).

It follows from Figure 11 that the quality of fitting experimental data with the theoretical model was fairly high, and the proposed chcracterization algorithm, being applied to experimental results, proved to be more efficient in comparison with the second derivative algorithm; see the data in Appendix B. Figure 12 shows the comparison of the mean relative errors *M* of depth prediction by using both techniques. In most cases, the NLF method provided relative errors of depth prediction under 15%, even if in some cases it reached 25–40%, which probably was associated with material imperfections and noise. In the case of the TSR algorithm, the characterization errors were 30–50%, and the maximal estimated depths were lower, because there were no distinct derivative peaks in certain experimental temperature curves. For example, the maximal estimated depth of the FBH (*D* = 3 mm) was 1.65 mm for the TSR method and 2.4 mm for the NLF method.

The results of defect characterization by using the NLF and TSR techniques in application to the experimental data are presented in Appendix B.

## 6. Conclusions

This study presents a new technique for characterizing defect depth in pulsed thermal NDT. The technique is based on the combination of a known simplified thermal NDT model and a non-linear fitting algorithm. It takes into account the finite lateral size of defects and the thermal reflection coefficient at the boundaries between the host material and defects, while the material apparent effusivity is to be determined experimentally. It was found that the model ensures a good fit between numerical and experimental data in the time range corresponding to the rising slope of temperature contrast evolutions. Therefore, the proposed method proved to be most efficient in the 0 < *t* < *t_pea_*_k_ time interval of growing temperature contrast.

The defect characterization efficiency was analyzed both numerically and experimentally on 3D-printed PLA samples with three types of defects (FBH, disks and spheres) of three dimensions placed at various depths. The results of numerical modeling demonstrated good agreement between the analytical and numerical models within the recommended time interval, thus resulting in fairly good accuracy of depth prediction by using the NLF technique. It was also demonstrated that changes in defect geometric shapes can be characterized by respective variations in the thermal reflection coefficient *R*; for example, depending on defect depth, *R* = 0.3–0.6 for spheres and 0.7–1 for disks and flat bottom holes. The relative errors of depth prediction by the proposed method applied to simulated data were 5–6%, while the TSR (second time derivation method) demonstrated errors of 10–25%. The same NLF methodology experimentally applied to 3D-printed PLA samples with low defect aspect ratios resulted in depth characterization errors of about 15% (25–40% in some cases), while the reference TSR method produced errors of 30 to 50%. The NLF method proved to be more efficient than the TSR technique in the evaluation of deeper defects.

The drawback of the proposed methodology is the dependence of results on heating non-uniformity that requires choosing a proper defect-free area. The characterization efficiency also depends on the accuracy of determined material thermal conductivity, apparent thermal effusivity and defect lateral size. Quantification of these limitations will be performed in future study.

## Figures and Tables

**Table 1:** Material thermal properties used in Comsol Multiphysics model.

Parameter	PAL	Air
Heat capacity (J/(kg∙K))	1800	1066
Thermal conductivity (W/(m∙K))	0.13	0.024
Density (kg/m^3^)	1240	1.276

**Table 2:** Sphere-like defects in 3D printed PAL samples.

Depth *d* (mm)		
*D* = 1 mm	*D* = 3 mm	*D* = 5 mm
0.3	0.6	1.5
0.45	0.9	1.8
0.6	1.2	2.1
0.75	1.35	2.25
0.9	1.5	2.4
1.05	1.65	2.55
1.2	1.8	2.7
1.35	2.1	3
1.5	2.4	3.3

## Appendix A

**Table A1:** Mean relative error of depth estimation by using TSR and NLF techniques (theory).

True Depth, *d_t_* (mm)	Depth Predicted by TSR, Second Time Derivative	$\mathrm{Relative}\;\mathrm{Error}\;\mathrm{of}\;\mathrm{TSR},\;\;\frac{d_{p}-d_{t}}{d_{t}}\times 100\%\;(\%)$	Depth Predicted by NLF	$\mathrm{Relative}\;\mathrm{Error}\;\mathrm{of}\;\mathrm{NLF},\;\;\frac{d_{p}-d_{t}}{d_{t}}\times 100\%\;(\%)$	*R* Measured by NLF
FBH *D* = 1 mm					
0.2	0.17	−17.15	0.18	−10.95	0.74
0.5	0.41	−18.84	0.46	−7.06	0.84
1	0.79	−21.13	0.96	−3.60	0.94
FBH *D* = 3 mm					
0.6	0.54	−9.82	0.59	−2.93	0.91
1.5	1.24	−17.11	1.45	−3.16	0.94
3	2.32	−22.62	2.88	−4.12	0.93
FBH *D* = 5 mm					
1	0.91	−9.26	0.99	−1.09	0.92
2.5	2.07	−17.32	2.39	−4.36	1
5	3.88	−22.32	4.74	−5.20	0.91
Disk *D* =1 mm					
0.2	0.16	−20.00	0.18	−11.8	0.73
0.5	0.41	−18.84	0.48	−4.54	0.90
1	0.79	−21.13	0.98	−2.50	1
Disk *D* = 3 mm					
0.6	0.55	−8.43	0.59	−2.40	0.87
1.5	1.23	−17.85	1.46	−2.58	0.99
3	2.31	−23.15	2.82	−5.86	0.79
Disk *D* = 5 mm					
1	0.90	−9.77	0.98	−2.37	0.81
2.5	2.04	−18.57	2.40	−3.98	0.85
5	3.80	−24.03	4.64	−7.19	0.62
Sphere *D* = 1 mm					
0.2	0.18	−11.80	0.19	−6.05	0.34
0.5	0.45	−10.28	0.52	3.92	0.53
1	0.87	−13.39	1.05	4.78	0.70
Sphere *D* = 3 mm					
0.6	0.54	−9.82	0.60	−0.60	0.39
1.5	1.30	−13.27	1.59	6.29	0.59
3	2.55	−15.17	3.09	2.85	0.62
Sphere *D* = 5 mm					
1	0.91	−8.76	0.99	−0.29	0.39
2.5	2.16	−13.68	2.68	7.17	0.60
5	4.24	−15.16	5.11	2.21	0.57

## Appendix B

**Table A2:** Mean relative error of depth estimation by using TSR and NLF techniques (experiment).

True Depth, *d_t_* (mm)	Depth Predicted by TSR, Second Time Derivative	Relative Error of TSR $\frac{d_{p}-d_{t}}{d_{t}}\times 100\%\;(\%)$	Depth Predicted by NLF	$\mathrm{Relative}\;\mathrm{Error}\;\mathrm{of}\;\mathrm{NLF},\;\frac{d_{p}-d_{t}}{d_{t}}\times 100\%\;(\%)$	*R* Measured by NLF
FBH *D* = 1 mm					
0.3	0.16	−46.10	0.29	−2.47	0.39
0.45	0.20	−55.55	0.59	30.47	0.93
0.6	0.40	−33.33	0.63	5.98	0.88
0.75	- *	-	0.76	1.87	0.53
0.9	-	-	1.05	16.78	0.84
1.05	-	-	1.18	11.92	0.76
FBH *D* = 3 mm					
0.6	0.37	−38.27	0.61	1.57	0.67
0.9	0.45	−49.99	0.92	2.39	0.77
1.2	0.85	−29.28	1.10	−8.53	0.67
1.35	1.01	−25.21	1.47	8.77	0.86
1.5	1.01	−32.69	1.49	−0.95	0.58
1.65	1.21	−26.52	1.60	−2.91	0.71
1.8	-	-	1.65	−8.55	0.50
2.1	-	-	1.91	−8.85	0.81
2.4	-	-	2.13	−11.35	0.46
FBH *D* = 5 mm					
1.5	0.80	−46.65	1.19	−20.77	0.58
1.8	1.47	−41.75	1.89	5.13	0.62
2.1	1.22	−41.84	2.02	−3.62	0.69
2.25	1.31	−18.54	1.86	−17.30	0.62
2.4	-	-	2.21	−8.02	0.69
2.55	-	-	2.32	−9.13	0.53
2.7	-	-	2.45	−9.19	0.60
3	-	-	2.80	−6.59	0.49
Disk *D* = 1 mm					
0.3	0.16	−46.1	0.42	39.97	0.43
0.45	-	-	0.57	25.80	0.57
0.6	-	-	0.70	17.17	0.65
0.75	-	-	0.81	7.76	0.44
0.9	-	-	1.13	25.82	0.69
1.05	-	-	1.23	17.33	0.46
Disk *D* = 3 mm					
0.6	0.40	−33.33	0.65	7.90	0.63
0.9	0.55	−38.42	1.02	12.97	0.32
1.2	0.89	−25.90	1.10	-8.29	0.54
1.35	1.01	−24.97	1.28	-5.13	0.53
1.5	1.15	−23.22	1.56	4.09	0.57
1.65	1.30	−21.16	1.60	−3.02	0.55
1.8	1.25	−30.43	1.90	5.72	0.68
2.1	-	-	2.22	5.90	0.52
2.4	-	-	2.14	-11.04	0.35
Disk *D* = 5 mm					
1.5	1.14	−23.79	1.57	4.65	0.69
1.8	1.36	−24.32	1.99	10.62	0.71
2.1	1.70	−19.26	2.12	1.16	0.56
2.25	1.71	−24.04	2.16	−3.88	0.51
2.4	-	-	2.20	−8.43	0.42
2.55	-	-	2.25	−11.62	0.44
2.7	-	-	2.38	−11.92	0.38
3	-	-	2.70	−10.00	0.45
Sphere *D* =1 mm					
0.3	-	-	-	-	-
0.45	0.26	−43.20	0.58	29.38	0.34
0.6	0.28	−53.33	0.62	3.25	0.42
0.75	0.34	−54.27	0.92	22.57	0.67
0.9	-	-	0.84	−6.31	0.47
1.05	-	-	1.00	−4.76	0.60
Sphere *D* = 3 mm					
0.6	0.40	−33.33	0.67	11.25	0.27
0.9	0.55	−39.08	1.14	26.86	0.24
1.2	0.77	−35.74	1.26	4.70	0.51
1.35	1.01	−24.97	1.37	1.29	0.49
1.5	1.09	−27.10	1.59	5.73	0.63
1.65	-	-	1.85	12	0.63
1.8	-	-	1.86	3.42	0.87
2.1	-	-	2.17	3.37	0.48
Sphere *D* = 5 mm					
1.5	0.87	−42.21	1.11	−26.15	0.26
1.8	1.01	−43.73	1.44	−19.87	0.25
2.1	1.25	−40.49	2.00	−4.54	0.30
2.25	-	-	1.75	−22.38	0.17
2.4	-	-	2.19	−8.83	0.27
2.55	-	-	2.10	−17.55	0.27
2.7	-	-	2.70	0.12	0.32
3	-	-	2.61	−13.16	0.27

*—No defect indication or too weak signal.

## References

[1] Ibarra-Castanedo C., Genest M., Servais P., Maldague X.P.V., Bendada A. (2007). Qualitative and quantitative assessment of aerospace structures by pulsed thermography. Nondestruct. Test. Eval..

[2] Shepard S.M. (2007). Flash Thermography of Aerospace Composites.

[3] Avdelidis N.P., Almond D.P., Dobbinson A., Hawtin B.C., Ibarra-Castanedo C., Maldague X. (2004). Aircraft composites assessment by means of transient thermal NDT. Prog. Aerosp. Sci..

[4] Yang B., Zhang L., Zhang W., Ai Y. Non-destructive testing of wind turbine blades using an infrared thermography: A review. Proceedings of the 2013 International Conference on Materials for Renewable Energy and Environment.

[5] Pilla M., Galmiche F., Maldague X.P., Maldague X.P., Rozlosnik A.E. Thermographic inspection of cracked solar cells. Proceedings of the SPIE 4710, Thermosense XXIV.

[6] Cotič P., Kolarič D., Bosiljkov V.B., Bosiljkov V., Jagličić Z. (2015). Determination of the applicability and limits of void and delamination detection in concrete structures using infrared thermography. NDT E Int..

[7] Hiasa S., Birgul R., Catbas F.N. (2016). Infrared thermography for civil structural assessment: Demonstrations with laboratory and field studies. J. Civ. Struct. Health Monit..

[8] Wiggenhauser H. (2002). Active IR-applications in civil engineering. Infrared Phys. Technol..

[9] Meola C., Boccardi S., Carlomagno G.M., Boffa N.D., Monaco E., Ricci F. (2015). Nondestructive evaluation of carbon fibre reinforced composites with infrared thermography and ultrasonics. Compos. Struct..

[10] Gholizadeh S. (2016). A review of non-destructive testing methods of composite materials. Proc. Struct. Integr..

[11] Yang R., He Y. (2016). Optically and non-optically excited thermography for composites: A review. Infrared Phys. Technol..

[12] Maldague X. (2001). Theory and Practice of Infrared Technology for Nondestructive Testing.

[13] Oswald-Tranta B. (2017). Time and frequency behaviour in TSR and PPT evaluation for flash thermography. Quant. Infrared Thermogr. J..

[14] Peeters J., Ibarra-Castanedo C., Sfarra S., Maldague X., Dirckx J.J.J., Steenackers G. (2017). Robust quantitative depth estimation on CFRP samples using active thermography inspection and numerical simulation updating. NDT E Int..

[15] Tang Q., Dai J., Liu J., Liu C., Liu Y., Ren C. (2016). Quantitative detection of defects based on Markov–PCA–BP algorithm using pulsed infrared thermography technology. Infrared Phys. Technol..

[16] Vavilov V.P., Burleigh D.D. (2015). Review of pulsed thermal NDT: Physical principles, theory and data processing. NDT E Int..

[17] Zeng Z., Li C., Tao N., Feng L., Zhang C. (2012). Depth prediction of non-air interface defect using pulsed thermography. NDT E Int..

[18] Švantner M., Muzika L., Houdková Š. Quantitative Inspection of Coatings Thickness by Time-Power Transformation Flash Pulse Thermographic Method. Proceedings of the 15th International Workshop on Advanced Infrared Technology and Applications (AITA 2019).

[19] Maldague X., Marinetti S. (1996). Pulse phase infrared thermography. J. Appl. Phys..

[20] Krapez J.-C., Lepoutre F., Balageas D. (1994). Early detection of thermal contrast in pulsed stimulated thermography. J. Phys. IV.

[21] Shepard S.M. (2007). Automated processing of thermographic derivatives for quality assurance. Opt. Eng..

[22] Almond D.P., Pickering S.G. (2012). An analytical study of the pulsed thermography defect detection limit. J. Appl. Phys..

[23] Sirikham A., Zhao Y., Mehnen J. (2017). Determination of thermal wave reflection coefficient to better estimate defect depth using pulsed thermography. Infrared Phys. Technol..

[24] Sun J. (2003). Method for determining defect depth using thermal imaging. U.S. Patent.

[25] Carslaw H.S., Jaeger J.C. (1959). Conduction of Heat in Solids.

[26] Almond D.P., Patel P., Patel P.M. (1996). Photothermal Science and Techniques.

[27] Parker W.J., Jenkins R.J., Butler C.P., Abbott G.L. (1961). Flash Method of Determining Thermal Diffusivity, Heat Capacity, and Thermal Conductivity. J. Appl. Phys..

[28] Boué C., Fournier D. (2009). Infrared thermography measurement of the thermal parameters (effusivity, diffusivity and conductivity) of materials. Quant. Infrared Thermogr. J..

[29] Almond D.P., Lau S.K. (1994). Defect sizing by transient thermography. I. An analytical treatment. J. Phys. D Appl. Phys..

[30] Marinetti S., Robba D., Cernuschi F., Bison P.G., Grinzato E. (2007). Thermographic inspection of TBC coated gas turbine blades: Discrimination between coating over-thicknesses and adhesion defects. Infrared Phys. Technol..

[31] Moskovchenko A., Vavilov V., Švantner M., Muzika L., Houdková Š. (2020). Active IR Thermography Evaluation of Coating Thickness by Determining Apparent Thermal Effusivity. Materials.

[32] Sd3d. PLA Technical Data Sheet. https://www.sd3d.com/wp-content/uploads/2017/06/MaterialTDS-PLA_01.pdf.

[33] Moskovchenko A.I., Vavilov V.P., Chulkov A.O. (2020). Comparing the efficiency of defect depth characterization algorithms in the inspection of CFRP by using one-sided pulsed thermal NDT. Infrared Phys. Technol..

[34] Sharath D., Menaka M., Venkatraman B. (2013). Defect Characterization Using Pulsed Thermography. J. Nondestruct. Eval..

[35] Grys S. (2018). Determining the dimension of subsurface defects by active infrared thermography—Experimental research. J. Sens. Sens. Syst..

